# Efficacy of semi-annual therapy of an extended-release injectable moxidectin suspension and oral doxycycline in *Dirofilaria immitis* naturally infected dogs

**DOI:** 10.1186/s13071-020-04380-z

**Published:** 2020-10-06

**Authors:** Bruno Alberigi, Julio I. Fernandes, Jonimar P. Paiva, Flavya Mendes-de-Almeida, Fabiana Knackfuss, Alexandre Merlo, Norma Labarthe

**Affiliations:** 1 Programa de Pós-Graduação em Medicina Veterinária, Universidade Federal Rural do Rio de Janeiro, Rio de Janeiro, Brazil; 2 Departamento de Medicina e Cirurgia Veterinária, Instituto de Veterinária, Universidade Federal Rural do Rio de Janeiro, Rio de Janeiro, Brazil; 3 Departamento de Patologia e Clínica Veterinária, Universidade Federal Fluminense - UFF, Niterói, RJ Brazil; 4 Universidade do Grande Rio - UNIGRANRIO, Duque de Caxias, RJ Brazil; 5 Technical Services for Companion Animals, Zoetis, São Paulo, Brazil; 6 Programa de Pós-Graduação em Bioética, Ética Aplicada e Saúde Coletiva. Escola Nacional de Saúde Pública, Fundação Oswaldo Cruz, Rio de Janeiro, Brazil

**Keywords:** Adulticide treatment, *Dirofilaria immitis*, Doxycycline, Heartworm, Macrocyclic lactone, Moxidectin

## Abstract

**Background:**

*Dirofilaria immitis* is a life-threatening nematode spreading globally. Arsenical treatment is currently recommended for removal of adult worms. However, arsenical treatment is not available in some countries, and there are dogs that cannot tolerate the rapid kill of adult worms; therefore, alternative adulticide slow-kill treatments are needed. Criticisms against the use of these alternative protocols include the potential for allowing disease to progress and for the development of ML-resistant worms.

**Methods:**

The efficacy of a protocol that includes semi-annual doses (i.e. every 6 months) of commercially available extended-release injectable moxidectin suspension (ProHeart^**®**^ SR-12) with 30-day oral administration of doxycycline was studied in 20 dogs with naturally occurring *D. immitis* infections. Each dog received treatment with ProHeart^**®**^ SR-12 (0.5 mg moxidectin/kg) by subcutaneous injection and oral doxycycline (10 mg/kg/bid × 30 days) every 6 months until two consecutive negative antigen test results were obtained. Pulmonary and cardiac evaluations were performed by radiographic and echocardiographic parameters. Physical examinations, complete blood counts, clinical chemistry profiles, microfilariae and antigen tests were performed periodically.

**Results:**

At enrollment, all dogs were positive for *D. immitis* antigen and 18 were microfilaremic. On day 30, microfilaremia counts decreased, and all dogs became amicrofilaremic by day 150. On day 180, 11 dogs were antigen-negative, and 7 more became negative by day 360. The two remaining antigen-positive dogs converted to negative by day 540 or 810. All antigen tests performed 180 days after the first negative test were negative. There was no decline in cardiac performance of the dogs throughout the study. Overall, pulmonary clinical conditions, presence of worms by echocardiography, and enlargement of caudal and main pulmonary arteries improved after treatment. Physical examinations, complete blood count results, and clinical chemistry profiles were within normal reference values. Respiratory conditions were improved, no damage to the heart was observed, and the treatment protocol was well tolerated by the animals.

**Conclusions:**

This alternative adulticide treatment was efficacious and well tolerated in naturally infected dogs. The injectable formulation provides the advantage of having veterinarians able to administer, monitor, and assess the efficacy and condition of the dog throughout the treatment and post-treatment periods.
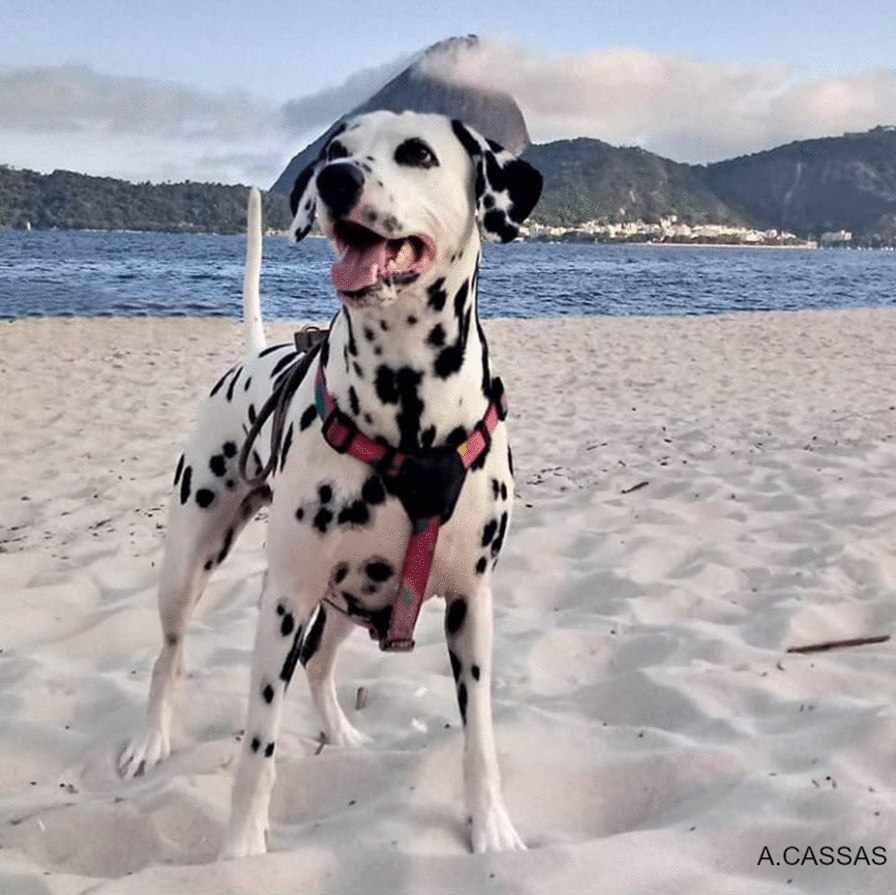

## Background

*Dirofilaria immitis* is a vector-borne filarial nematode that infects dogs around the world. The number of infected dogs and the areas of the parasite distribution are increasing [1, 2]. This increase is thought to be a consequence of the movement of infected dogs as well as global climate changes, which favor the spread of vector mosquito species [1, 3–5]. Heartworm disease in dogs is progressive and potentially life-threatening, as it primarily affects the heart and lungs. Therefore, treatment should target the elimination of all forms of the parasite as soon as possible with minimal treatment-related adverse effects. This will minimize progression of the lesions [2].

The recommended treatment protocol includes administration of the adulticidal melarsomine with a macrocyclic lactone (ML) and doxycycline [2, 6]. An alternative adulticide protocol that limits administration to only doxycycline and a ML is accepted only when melarsomine is contraindicated or unavailable [6–8]. The adulticidal effect of MLs was first studied using ivermectin or milbemycin oxime at prophylactic doses [9]. Both drugs were shown to partially reduce the number of young adult worms when initiated four months after experimental infection and continued for 14 monthly doses. Alternative protocols combining moxidectin or ivermectin topical or ivermectin oral and doxycycline, with some combinations also adding 10% imidacloprid, have been shown to be effective [4, 10–15].

Moxidectin was selected for testing here primarily due to its higher intrinsic potency against filarial nematodes when plasma concentrations provide a longer parasite exposure to the drug compared with other ML, including against ML-resistant heartworm strains [16–18]. Pharmacokinetic characteristics of moxidectin, including its high distribution in lipid tissues, reduced metabolism, and long half-life, contribute to its accumulation and high potency [18, 19].

To consider an animal free of infection when using an alternative protocol, two consecutive negative antigen tests must be obtained six months apart [20]; therefore, the treatments must be administered according to the prescribed schedule for at least 12 months. Since non-compliance during monthly administration of treatments over long periods of time is known to be an issue by negatively influencing outcomes [20, 21], long-acting formulations have been developed to improve compliance and convenience for pet owners.

An extended-release injectable moxidectin suspension formulation (ProHeart^**®**^ 12/ProHeart^**®**^ SR-12, Zoetis, Parsippany, NJ, USA) contains 1% moxidectin microspheres after constitution according to product instructions and is designed to deliver 0.5 mg/kg moxidectin. This product prevents heartworm disease caused by *D. immitis* for 12 months in dogs after a single injection. ProHeart^**®**^ 12 and ProHeart^**®**^ SR-12 are brand names of the same extended-release microsphere formulation of moxidectin, the former being marketed in the USA and the later in Australia, Japan, southeast Asia and Brazil. Recently published studies report the efficacy of a single administration of injectable moxidectin at 0.5 mg/kg (1 ml/20 kg) by subcutaneous (SC) injection as a heartworm preventative and safety of 1×, 3× and 5× the dose every six months for three treatments in normal beagles beginning at six months of age and as high as 5× the use rate in reproducing animals, ivermectin-sensitive dogs, and heartworm-positive dogs [16, 22].

Due to the unavailability of melarsomine-based products in Brazil and taking into consideration the increased prevalence of heartworm infection in the country [23, 24], the aim of the present study was to evaluate the efficacy of a heartworm-preventive protocol that includes the commercial formulation of an extended-release injectable moxidectin suspension (ProHeart^**®**^ SR-12) administered every six months plus cycles of 30-day treatment with oral doxycycline in dogs naturally infected with *D. immitis.*

## Methods

### Selection criteria

Following submission of owner’s consent for their animals to participate in the study, 20 dogs testing positive for adult *D. immitis* antigens (SNAP^®^ 4DX Plus^®^, IDEXX Laboratories Inc., Westbrook, ME, USA) were eligible to participate in the study. Dogs had to be 1–10 years-old, be clinically healthy, have a platelet count > 150 × 10^3^/µl, and be free of left ventricular function disorders and severe concomitant diseases. There were no size or sex restrictions, but some breeds (collies, shelties and Australian shepherds), females at any stage of pregnancy, and dogs that had been on doxycycline or MLs in the past 6 months were not eligible for enrollment.

### Study subjects

The 20 dogs selected to participate in the study included 11 females and 9 males and most were neutered (6 females and 7 males). Mean age was 4.85 years (range: 1–8 years). The majority of the dogs (*n* = 13) were mongrel. Purebred dogs included miniature pinscher, Teckel (also known as dachshund), Brazilian terrier, Dalmatian, Staffordshire bull terrier, and Labrador retriever.

### Treatments

**Fig. 1:**
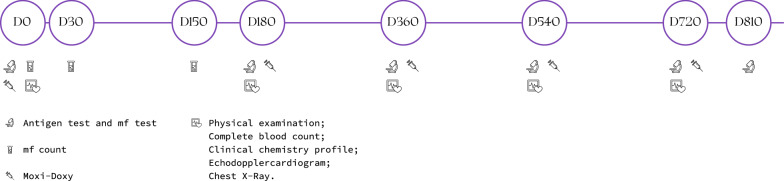
Treatment schedule with ProHeart® SR-12 associated with oral doxycycline (Moxi-Doxy) and evaluations

Each dog received a treatment cycle consisting of a single administration of extended-release injectable moxidectin suspension (ProHeart^**®**^ SR-12; Zoetis, Olot, Spain) 0.5 mg moxidectin/kg and doxycycline administered orally at 10 mg/kg bid for 30 days every 6 months ± 5 days after the first dose on day 0. This treatment regimen was repeated and recorded on the individual data capture form by the investigator until the dog was considered cleared of the infection (two consecutive antigen negative tests using unheated samples obtained 6 months apart) as recommended by the American Heartworm Society (Fig. 1) [2]. All dogs were weighed before each treatment, and all injections of extended-release injectable moxidectin suspension were performed by the same veterinarian. Doxycycline was administered orally to the dogs at home by dog owners. The dogs were kept in their natural environment by their owners and managed according to their routine by the owner. Although exercise was not strictly restricted, owners were instructed to avoid excess of physical activities.

### Efficacy evaluations

On day 0, dogs were tested for the presence of adult *D. immitis* antigens (SNAP^®^ 4DX Plus^®^, IDEXX Laboratories Inc., Westbrook, ME, USA) according to the procedure provided by the manufacturer, and microfilariae counts were performed using a modified Knott’s test [12]. On the same day, a clinical examination, echocardiogram, and chest radiographs also were performed.

All tests and examinations performed on day 0 were repeated every 6 months (± 5 days), except microfilariae counts, until the dog was considered free of the heartworm infection (two consecutive antigen negative tests using unheated samples 6 months apart). Microfilariae counts after day 0 were performed on days 30 and 150 (± 5 days). In order to compare the antigen results of preheated and unheated samples, when a dog tested negative the sample was preheated and retested [12, 25].

Pulmonary and heart silhouette evaluations were always performed by the same veterinarian by chest radiographs in three positions: ventrodorsal and laterolateral for both sides. The echocardiogram was always performed by the same veterinarian to evaluate the presence of worms in the pulmonary artery and to assess right ventricular function. Systolic right ventricular function was evaluated using fractional variation of the right ventricular area, tricuspid annular plane systolic excursion, and S-wave velocity. The diastolic right ventricular function was evaluated using transtricuspid flow and right ventricular tissue Doppler imaging. Pulmonary arterial pressure was evaluated using velocity of turbulent flow of tricuspid valve regurgitation, pulmonary artery distensibility index, right atrioventricular silhouette enlargement, and the presence of tricuspid murmur.

### Safety evaluations

Since extended-release injectable moxidectin suspension was administered every 6 months, instead of annually as recommended for use as a heartworm preventative on the product label, physical examinations and blood tests (complete blood count and clinical chemistries) were performed to evaluate potential adverse reactions at days 0, 180, 240, 360, 540 and 720 (± 5 days) while dogs were still undergoing treatment (Fig. 1).

### Statistical methods

The individual dog was the experimental analysis unit. The geometric means of microfilariae counts post-treatment were compared with counts on day 0 by Student’s t*-*test. The pulmonary parameters were tested by a Chi-square test with Yates correction when appropriate. To be considered significant, at least 50% of the measurements of cardiac functions should differ from results recorded on day 0. The comparison of blood test values was performed by the Friedman test. All tests considered 5% or 1% levels of significance using IBM^®^ SPSS v25.0.

## Results

### Parasitological evaluation

At enrollment, 18 of the dogs were microfilaremic, and the geometric mean ± standard deviation (SD) of microfilariae counts of these dogs was 4587 ± 8.6 mf/ml. One dog became amicrofilaremic on day 30, and the geometric mean of microfilariae counts of the other 17 microfilaremic dogs decreased to 2584 ± 7.2 mf/ml by day 30 (*t* = 2.3487, *df* = 19, *P* = 0.03). On day 150, all animals were amicrofilaremic (Table 1).

**Table 1:** Microfilariae count and number of *D. immitis* antigen-positive dogs treated with Moxi-Doxy

Day	Microfilariae		Antigen test results		
	No. positive	Mf/ml	No. positive	No. negative 1st test	No. negative 2nd test
0	18	4587^a^	20	0	0
30	17	2584^b^	nd	nd	nd
150	0	0	nd	nd	nd
180	0	0	9	11	0
360	0	0	2	7	11
540	0	0	1	1	7
720	0	0	1	0	1
810	0	0	0	1	0

*Notes*: Moxi-Doxy, semi-annual treatments with extended-release injectable moxidectin and oral doxycycline. Different letters in column indicate significant difference (*P* < 0.05)

*Abbreviations*: Mf/ml, geometric mean microfilariae/ml of blood; Day 0, before treatment; nd, not done

All dogs were antigen-positive using unheated samples on day 0. Eleven dogs became antigen-negative on day 180; 7 dogs became antigen-negative on day 360, and 1 dog on day 540 (Table 1). All antigen tests performed 180 days after their first negative antigen test were negative. On day 540, one dog was still antigen-positive; therefore, treatment was continued for this dog. The dog was re-checked on day 810, when the antigen test became negative for the first time. Antigen testing was not available for that dog after the first negative antigen test. Therefore, only results of the microfilariae analysis are provided for that dog.

There was some disagreement between unheated and preheated results when the first antigen-negative test result was obtained; only 11 of the 19 results (58%) showed agreement. Of these, 6 were dogs testing negative on day 180 and 5 that converted to negative by day 360. All the second unheated antigen-negative results agreed with the preheated results. There was no agreement between the unheated and preheated results of the first negative test on day 540.

### Pulmonary evaluation at first unheated sample negative antigen test

Overall, treatment with extended-release injectable moxidectin suspension and doxycycline improved most pulmonary criteria at evaluations performed at the time of the first negative antigen test (Table 2). At the first unheated sample negative antigen test, the number of dogs with pulmonary signs decreased significantly compared with the number observed on day 0, including cough (1 *vs* 7; *P* < 0.0001); dyspnea (1 *vs* 3; *P* = 0.028); harsh expiratory sounds (11 *vs* 16; *P* < 0.0001); presence of worms in the main pulmonary artery (3 *vs* 7; *P* = 0.0013); enlargement of the main pulmonary artery (4 *vs* 8; *P* = 0.023), and enlargement of the caudal pulmonary artery (5 *vs* 8; *P* = 0.0275). The number of dogs presenting micronodular pattern increased from the number observed on day 0 (9 *vs* 6; *P* = 0.0324), and the number of dogs showing bronchial and interstitial pattern at the first negative antigen test was unchanged relative to the number observed on day 0.

**Table 2:** Number of *Dirofilaria immitis-*infected dogs treated with Moxi-Doxy according to pulmonary evaluation and antigen detection

Pulmonary evaluation	No. of dogs (%)		
	Day 0	First negative antigen test	Second negative antigen test
Cough	7 (36.8)^a^	1 (5.3)^b^	1 (5.3)^b^
Dyspnea	3 (15.8)^a^	1 (5.3)^b^	0 (0)^b^
Harsh expiratory sounds	16 (84.2)^a^	11 (57.9)^b^	2 (10.5)^c^
Worm present at echocardiography	7 (36.8)^a^	3 (15.8)^b^	0 (0)^c^
Caudal pulmonary artery enlargement	8 (42.1)^a^	5 (26.3)^b^	2 (10.5)^c^
Main pulmonary artery enlargement	8 (42.1)^a^	4 (21.1)^b^	3 (15.8)^b^
Bronchial + interstitial pattern	19 (100)	19 (100)	18 (94.7)
Micronodular pattern	6 (31.6)^a^	9 (47.4)^b^	7 (36.8)^a^

*Notes*: Moxi-Doxy, semi-annual treatments with extended-release injectable moxidectin and oral doxycycline. Different letters in rows indicate significant differences (*P* < 0.05)

### Pulmonary evaluation at second unheated sample negative antigen test

When the number of dogs presenting pulmonary signs on the second negative antigen test was compared with those on day 0, most pulmonary variables were relatively consistent with findings at the first negative antigen test (Table 2). However, at this evaluation, the number of dogs with harsh expiratory sounds (2 *vs* 16; *P* < 0.0001), worm presence at echocardiography (0 *vs* 7; *P* < 0.0001), and caudal pulmonary artery enlargement were significantly less than on day 0 (2 *vs* 8; *P* < 0.007); however, the number of dogs showing micronodular pattern at the second negative antigen test was statistically similar to that observed day 0 (7 *vs* 6; *P* = 0.052), and the number of dogs presenting bronchial and interstitial patterns was essentially the same as on day 0 (Table 2).

### Cardiac evaluation

Systolic right ventricular function evaluation was within the normal limits throughout the study, and showed virtually no change from day 0. Signs of pulmonary hypertension showed no evidence of worsening throughout the study, and improvement was observed for one dog at the second negative antigen test.

Although there are no normal standards for evaluation of diastolic right ventricular function evaluation, improvement was noted for 11 dogs at the time of the first negative antigen test. At the second negative antigen test, improvement was noted for two more dogs.

### Safety evaluation

Dogs received from 2 to 5 doses of the extended-release injectable moxidectin suspension every 6 months, and even after 5 semi-annual doses with extended-release injectable moxidectin suspension, there was no evidence of adverse effects on body weight or clinical health of any dog in the study. There were no local reactions at the injection site observed for any dog.

Results of clinical chemistry and complete blood count variables were all within normal reference values throughout the study. Comparison of results at sampling times during the treatment period with results from day 0 showed no variation or treatment-related increases in kidney or liver serum biochemical blood tests, including BUN, creatinine, alanine aminotransferase, and alkaline phosphatase.

## Discussion

The parasitological results of the present study showed that the association of an extended-release injectable moxidectin suspension formulation (0.5 mg/kg moxidectin) administered off label (semi-annual *vs* annual administration) with a course of oral doxycycline was able to eliminate *D. immitis* infections with no apparent treatment-related adverse effects to the animals. Regardless of the time needed to clear antigens, the concentration of microfilariae declined significantly within 30 days of initiation of treatment. Complete elimination of microfilariae for all 20 dogs was ≤ 150 days, requiring only a single treatment. In a previous study of the same extended-release moxidectin suspension in dogs experimentally infected with D. *immitis*, microfilaremia decreased rapidly, with an average reduction of 20.9% one day after treatment and 96.9% on day 84, although this study used a ML-resistant strain [17]. In a study conducted as part of the pivotal safety evaluation of ProHeart^**®**^ 12, a 3× dose (1.5 mg/kg) demonstrated a > 99% reduction in geometric mean microfilaria counts on day 15 post-treatment against a susceptible strain of heartworm [22].

In a study of the efficacy and safety of the extended-release injectable moxidectin suspension administered every six months with a 30-day course of doxycycline in a female dog rescued from the streets, microfilariae were not detected when the dog was presented for the second moxidectin treatment on day 180 [26]. The speed of elimination of microfilariae observed in the present study is comparable to results reported for monthly administration of topical moxidectin/imidacloprid and 30 days with oral doxycycline [4, 10, 27].

The use of doxycycline has been shown to improve post-treatment complications following adulticide treatment with melarsomine [7]. Doxycycline is included as a contributor in the alternative adulticide treatment protocol to reduce the worms’ fitness and the host’s lung tissue inflammation as a consequence of its action on the endosymbiont *Wolbachia* [6–8]. Doxycycline given alone gradually reduces numbers of microfilariae and impairs the development of immature forms of *D. immitis* resulting in their inability to infect new definitive hosts. This effect is likely to be effective in reducing the rate of selection of *D. immitis* with genes that confer resistance to MLs [28].

Since all negative results at the first antigen testing at six months after initial treatment were still negative at the second test six months later, it can be assumed that adult worms were eliminated even before the first negative test result was obtained, especially because antigen levels are known to decline slowly after worm death [4]. Therefore, results indicate that 90% of the dogs were free of adult worms within one year of treatment.

The elimination of adult worms obtained using an extended-release injectable moxidectin suspension formulation and oral doxycycline in this study were similar to those reported for treatments using 10% imidacloprid + 2.5% moxidectin together with doxycycline [4, 27]. Since these earlier studies and the present study included naturally infected pet dogs, there is no information on worm burden or duration of infection; therefore, the subtle differences observed among the results of the present study and those from the earlier studies do not necessarily reflect differences in activity or efficacy.

Preheating is known to enhance the test’s sensitivity for specific *D. immitis* antigens; however, it reduces specificity, leading to cross-reactions with other parasites [29]. In the present study, preheating the samples was shown to be unnecessary once preheated and unheated samples tested negative when the dog was considered free of the infection. A preheated sample showed a positive antigen result in disagreement with the initial unheated result; however, in a follow-up pair of tests, both the unheated and heated tests confirmed the first negative result. Therefore, as reported before, the need for sample preheating to confirm treatment success is debatable [25], as the need for two consecutive unheated negative results to consider a dog free of infection [15].

The pulmonary conditions of the dogs at the time they were enrolled in the study showed that lungs were clearly affected by the infection. There are arguments against the use of alternative treatment protocols because it takes longer than the arsenical treatment to eliminate worms, allowing the lesions to progress [2, 30]. However, in the present study, fewer dogs were positive for signs of cardiopulmonary disease, including cough, dyspnea, harsh expiratory sounds, presence of worms detected by echocardiography, and enlargement of caudal and main pulmonary arteries at the time the first negative antigen test than were diagnosed before treatment initiation. This difference became even more evident at the second negative antigen test, showing that although lung lesions may be persistent once that have become established [31], the time needed for the treatment to kill the worms was short enough to prevent worsening of the majority of respiratory conditions.

Nearly all dogs presented interstitial and bronchial patterns on day 0 and throughout the study, demonstrating that the lung inflammatory reaction may have turned into fibrosis during the infection [31]. The increase of the number of dogs showing a micronodular pattern on the day of the first negative antigen test suggests that the unavoidable thromboembolism due to worm death caused the acute inflammation [32–34]. It is interesting to note that the frequency of dogs presenting signs of acute inflammation at the second negative antigen test decreased, suggesting that the lesion tended to heal overtime.

The treatment did not negatively affect cardiac performance. At enrollment, all dogs presented a favorable cardiac evaluation, with no signs of heart failure even though they had active heartworm infection, which would predispose them to this condition [35, 36]. The systolic function of the right ventricle throughout the study showed that the alternative treatment used did not culminate in *cor pulmonale* as has been previously reported [4, 27]. The conservation of the systolic function of the right ventricle in association with the lack of variation in the pulmonary pressure assessments throughout the treatment are encouraging. Although the death of adult worms causes thromboembolism [2, 34], the dogs in the present study showed no signs of disease due to thromboembolism even when tested after the first negative antigen test. This observation suggests that there is a slower rate of death of worms with the treatment protocol in this study compared with rapid kill provided with administration of arsenical [4], which could provide a valuable safety benefit. The long-term effects of killing heartworms slowly using MLs is not completely clear, and some have argued that this practice is even undesirable, as it may allow disease to progress during the extended “slow kill” process and additionally, potentially increase the risk of ML-induced resistance [37, 38]. However, co-administration of doxycycline along with the potent microfilaricidal efficacy observed with extended-injectable moxidectin suspension, even against ML-resistant heartworms, as well as reduced compliance risk, would seem to mitigate the risk of additional resistance effects [17].

Little is known about canine diastolic right ventricle function, and information on how heartworm infection affects diastolic right ventricle is even scarcer [39]. As 68% of the dogs in the present study showed improvement in diastolic function following completion of the treatment protocol, it can be inferred that diastolic function had been directly affected by heartworm disease. The results suggest that diastolic right ventricle function of heartworm-infected dogs must be addressed and monitored throughout adulticidal treatment.

The plasma levels of an extended-release injectable at 0.5 mg/kg has been reported, indicating that these plasma levels of moxidectin decline over the year after a single dose, which then indicates the need for an annual injection to ensure that dogs are properly protected according to the product label for heartworm prevention [40]. Based on a possible reduction in microfilaricidal and adulticidal effects due to lowering of plasma or tissue moxidectin levels with this yearly dosing interval, for this study it was decided to adopt an off-label protocol consisting of semi-annual doses of the extended-release moxidectin formulation. Although pharmacokinetics of moxidectin in such a protocol were not evaluated in the present study, results show that administration of moxidectin semi-annually was acceptable in terms of safety. Treatment-related adverse effects were not reported by dog owners or treating veterinarians, and hematologic and biochemical parameters were maintained within reference ranges throughout the treatment. Results of previous studies have shown that administration of up to 5× the label dose of 0.5 mg/kg of extended-release injectable moxidectin suspension, administered every six months for three consecutive treatments, was well tolerated [22]. Additional studies should be conducted to establish safety of the combined protocol with moxidectin plus doxycycline; however, from a clinical perspective, the treatment protocol followed in this study was shown to be effective and well tolerated in these pet dogs with naturally occurring heartworm infections. While “slow-kill” protocols are not recommended by the American Heartworm Society [2], in areas such as Brazil where heartworm prevalence is substantial and no arsenical adulticide products are available, alternate protocols such as the one reported here should be considered for compassionate use under veterinary discretion to provide heartworm-infected dogs treatment from this devastating disease. It should be noted that neither ProHeart^**®**^ SR-12 nor ProHeart^**®**^ 12 are indicated for the treatment and removal of adult heartworms and the protocol followed in this study is considered an “off label” use of the product.

## Conclusions

Significant decreases in microfilaremia occurred within 30 days, respiratory conditions were improved, and the majority of dogs were free of infection approximately one year following administration of semi-annual doses of extended-release injectable moxidectin suspension plus 30-day oral treatment with doxycycline to dogs naturally acquired *D. immitis* infections. The alternative heartworm adulticide protocol also was well tolerated by all animals.

## Data Availability

The datasets used and/or analysed during the present study are available from the corresponding author on reasonable request.

## Abbreviations

ProHeart® SR-12: Extended-release injectable moxidectin suspension
ML: Macrocyclic lactone
BUN: Blood urea nitrogen
Moxi-Doxy: Extended-release injectable moxidectin and oral doxycycline
Mf/ml: Geometric mean microfilariae/ml of blood
Nd: Not done
Mfs: Microfilariae
